# Building bridges between brain and behavior: An open-source toolbox for joint modeling with fMRI

**DOI:** 10.1162/IMAG.a.1272

**Published:** 2026-06-15

**Authors:** Niek Stevenson, Steven Miletić, Birte U. Forstmann

**Affiliations:** University of Amsterdam, Amsterdam, The Netherlands; Leiden University, Leiden, The Netherlands

**Keywords:** joint modeling, fMRI, hierarchical Bayes, evidence accumulation models, diffusion decision model

## Abstract

Understanding how neural activity relates to behavior remains a central challenge in cognitive neuroscience. Joint modeling offers a principled method by simultaneously fitting behavioral and fMRI data and estimating the relations between them, accounting for measurement error, inter-individual variability, and shared uncertainty. Here, we present a joint modeling toolbox built in the *R* package, **EMC2**, which streamlines the behavioral, neural, and joint model estimation. We apply the toolbox to a perceptual decision-making task performed in an fMRI scanner, demonstrating how to specify behavioral and neural design matrices, set priors, construct brain–behavioral links, and conduct group-level tests and individual-difference analyses. Through the hierarchical modeling framework, joint estimation stabilizes weakly identified parameters and mitigates attenuation bias in brain–behavior correlations. Our toolbox, accompanied by practical code examples, provides an easily accessible introduction for researchers to adopt joint models and to derive richer, more reliable insights into the cognitive and neural mechanisms underlying human behavior.

## 1 Introduction

The fundamental challenge of cognitive neuroscience is to unravel the neural mechanisms underlying human cognition, linking complex mental processes to brain function (Schall, 2004; Teller, 1984). To that end, researchers measure how experimental manipulations affect brain activity and behavior. Advances in neuroimaging methods such as functional magnetic resonance imaging (fMRI) have enhanced our ability to measure neural activity with increasing spatial and temporal precision (Feinberg et al., 2023). Simultaneously, increasingly sophisticated computational models of behavior are used to translate psychological theories into testable frameworks (Heathcote & Matzke, 2022; Lewandowsky & Farrell, 2011).

Taken together, these advances increasingly facilitate the study of the latent cognitive states that jointly generate behavioral and neural observations. Testing such linking propositions calls for statistical models that embed explicit bridging functions between brain and behavior (Teller, 1984; Turner et al., 2019). However, inference on the relationship between behavior and brain activity is often based on separate analyses that are linked post hoc. Moreover, such a two-step approach ignores the substantial measurement error present in both behavioral and neural data (e.g., due to attention lapses or physiological noise; Chen et al., 2017; Rouder & Haaf, 2019). Consequently, the estimated relationships are diluted by measurement error and biased toward zero (Matzke et al., 2017; Spearman, 1904).

The search for bridging functions have motivated the development of joint models that unify the behavioral and neural levels in a single framework (Ghaderi-Kangavari et al., 2023; Kang et al., 2022; Miletić et al., 2025; Nunez et al., 2024; Palestro et al., 2018; Stevenson, Innes, Gronau et al., 2024; Turner et al., 2015, 2016, 2019). By estimating all parameters simultaneously, joint models account for measurement error in both domains, constructing a statistically principled and unbiased bridge that is mutually informed by the behavioral and neural data (Turner et al., 2013).

Despite the above-described advances, applications of joint neural-behavioral models with fMRI data have remained relatively limited. We believe this is mostly due to two reasons. First, on top of the already high bar of understanding the neuroimaging process as well as the psychological and neuroanatomical background of the hypothesis, joint modeling requires expertise in cognitive modeling, neural modeling, and multi-level (hierarchical) statistics. Second, although some tutorials exist (Palestro et al., 2018; Turner et al., 2019, 2016), they require users to have a working knowledge of probabilistic programming languages. Third, joint models constitute highly dimensional Bayesian models that can be extremely computationally expensive to estimate using generic software (Stevenson, Innes, Boag, et al., 2024).

To address these challenges, we created a Bayesian joint modeling toolbox in the *R* package **EMC2** (Stevenson, Donzallaz, et al., 2024). **EMC2** uses straightforward syntax to facilitate construction of models and leverages recent advances in model estimation, making it possible to estimate neural-behavioral joint models, even on a personal computer. **EMC2** supports a wide range of user-friendly design and prior specification functions and plots, as well as model-fit assessment and model comparison metrics. Here we focus on how to implement joint fMRI–behavioral models; for a tutorial on joint modeling with EEG/MEG, we refer the reader to Nunez et al. (2024). We start by providing an overview of the different components of a joint model, including the cognitive model, fMRI model, and the link between the two.

To illustrate the joint modeling framework, we will use a hypothetical example experiment where a researcher is interested in the behavioral and neural correlates of the effect of task difficulty. To that end, the researcher designs an experiment, performed in an fMRI scanner, that requires participants to make perceptual decisions. There are both “Easy” and “Hard” trials in the task. These conditions are expected to differ in both cognitive processes and neural responses. The dependent variables of interest are the choices and response times, and the blood oxygen level-dependent (BOLD) responses in a variety of brain regions of interest. We begin by outlining modeling approaches for behavioral and neural data separately, before introducing the hierarchical joint models in detail. Afterward, we will apply the joint modeling framework to re-analyze existing, openly accessible fMRI data.

## 2 Cognitive Modeling

Conventional statistical analyses of behavioral data often focus on describing empirical patterns in observed (manifest) data, such as testing whether mean response time or choice accuracy differs between conditions. While useful for establishing the existence of an effect, these standard statistical models are purely descriptive; they do not explain *how* or *why* the behavior occurred (Lewandowsky & Farrell, 2011).

In contrast, mechanistic, process-based cognitive models formalize psychological theories into mathematical equations that represent the hypothesized cognitive processes generating the behavior. Rather than only describing the data, cognitive models use parameters that offer direct insights into latent cognitive components.

This distinction is particularly important for neuroscientific research. Brain regions and neural networks are thought to implement specific cognitive operations rather than broad behavioral outcomes. By decomposing behavior into its constituent latent processes, researchers can map neural signals onto the algorithmic steps of cognition (Turner et al., 2019).

Different classes of cognitive models exist, such as multinomial processing tree models (Batchelder & Riefer, 1999; Erdfelder et al., 2009), signal detection theory (Green & Swets, 1966; Macmillan & Creelman, 2004), reinforcement learning models (Dayan & Niv, 2008; Sutton & Barto, 2018), and evidence accumulation models (Brown & Heathcote, 2008; Ratcliff, 1978).

In this paper we will use evidence accumulation models as the cognitive model part of the joint model.

EAMs are a class of cognitive models that are uniquely suited for analyzing speeded decisions. Independent analysis of choice outcomes and response times ignores the dynamic relationship between both brain and behavior. Observers can flexibly alter their behavior, choosing to respond quickly but with lower accuracy, or slowly but with higher accuracy (Rae et al., 2014; Ratcliff, 1978; Stone, 1960). EAMs overcome this problem by jointly modeling the full distributions of choices and response times. Breaking down manifest behavior into distinct latent cognitive operations that can be mapped onto separate neural signals. Although multiple variants exist, evidence accumulation models (EAMs) assume that decisions arise from the accumulation of evidence over time until a response threshold is reached (Brown and Heathcote, 2008; Donkin & Brown, 2018; Ratcliff et al., 2004, 2016). This process is characterized by three core parameters: the drift rate (v), indexing the mean speed of information processing; the response threshold (a), governing the speed–accuracy trade-off; and the non-decision time ($t_{0}$), capturing stimulus encoding and motor execution.

**Fig. 1.**
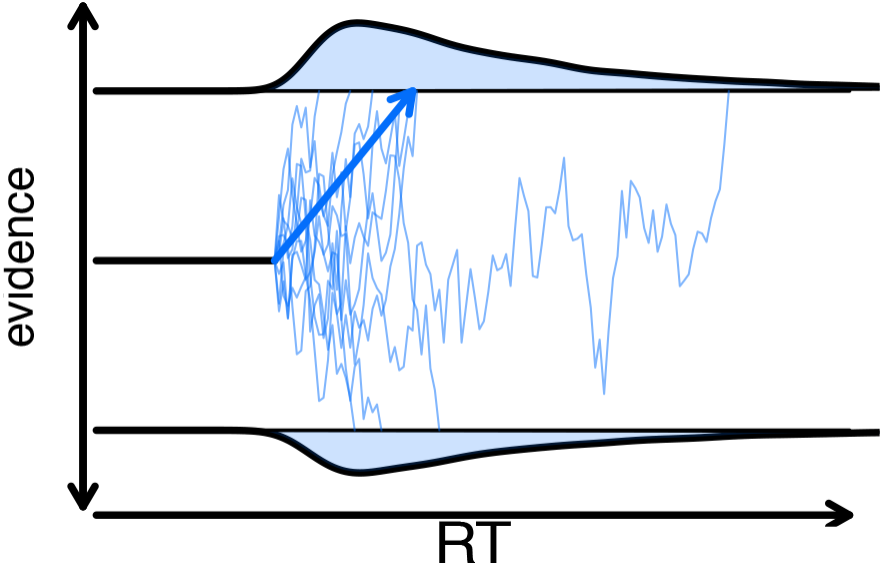
The diffusion decision model (DDM). An evidence accumulator (blue line) accrues evidence over time (y-axis) until one of two decision boundaries (black horizontal lines) is crossed, with separation between them (response boundary a) representing response caution. The thick blue line represents the mean slope of the evidence accumulation process (drift rate v), which is subject to random noise. The individual lines represent the evidence accumulation on different trials. Once the accumulated evidence crosses either boundary, the decision is made. The response time (RT) is the time it takes for the evidence to cross the boundary, plus the non-decision time ($t_{0}$), which accounts for stimulus encoding and motor execution time. The RT distributions for the two choices are plotted as density distributions in the blue shaded areas outside the boundaries.

In our example experiment, the decision task was a two-alternative forced choice task. The researcher uses the most prominent evidence accumulation model: the diffusion decision model (DDM; Fig. 1; Ratcliff, 1978).

The DDM has a single noisy evidence accumulator accumulating competing evidence for two choices associated with the upper and lower boundaries. The mean speed of evidence accumulation is governed by the drift rate. The boundary separation represents response caution. Once the evidence trajectory surpasses one of the boundaries, the associated response is triggered. The response time is the time it takes for the evidence to cross the boundary, plus the non-decision time ($t_{0}$), which accounts for stimulus encoding and motor execution time.

A common finding is that task difficulty affects the drift rate: harder trials slow evidence accumulation (Ratcliff et al., 2004). To that end we want to capture drift rate differences between conditions. An intuitive approach would be to first estimate the drift rate based on the easy trials, then the drift rate based on the hard trials, and subsequently compare the two by taking their difference. However, recall that the goal of joint modeling is to relate this *difference* measure to neural data. As such, the researcher directly estimates the between-condition difference in drift rates as a parameter, alongside the mean drift rate across conditions. Linking these difficulty parameters within our joint model allows us to assess their relationship.

To that end, we construct a design matrix that parameterizes the drift rate using an intercept and a contrast between the easy and hard conditions:

**Table:** 

			Design Matrix	
Trial	Onset (s)	Trial Type	Intercept	Contrast
1	10	Easy	1	-0.5
2	20	Hard	1	0.5
3	35	Hard	1	0.5
4	42	Easy	1	-0.5
5	60	Hard	1	0.5
6	75	Easy	1	-0.5

The drift rate (v) for trial l is a single value modeled as a linear combination of the design covariates:

$$v_{l}=v_{0}+x_{contrast_{l}}v_{\text{dif}},$$ (1)

where $x_{contrast_{l}}$ denotes the design matrix entry for trial l, $v_{0}$ is an estimated parameter that encodes the intercept drift rate (i.e., the mean drift rate), and $v_{\text{dif}}$ is the estimated parameter for the difference in drift rate between the two conditions. The researcher assumes that the boundary separation (a) and non-decision time ($t_{0}$) parameter are unaffected by the difficulty manipulation, and are modeled using only an intercept.

## 3 Modeling of fMRI Data

With the cognitive model in place, we next outline the steps typically taken to model the BOLD signal. As with the behavioral model, the researcher is interested in the effect difficulty has on the BOLD signal. To that end, a conceptually similar design matrix that mapped the experimental design to the drift rate can also be used to map regressors to the BOLD signal using a general linear model (GLM):

$$y_{rv}=x_{intercept}\beta_{0_{r}}+x_{contrast_{v}}\beta_{{\text{dif}}_{r}}+\epsilon_{rv},$$ (2)

with $y_{rv}$ the BOLD signal time course for region r, at volume v, $\beta_{0_{r}}$ the intercept parameter, $\beta_{{\text{dif}}_{r}}$ the difference parameter, and $\epsilon_{rv}$ a residual noise term, capturing residual variability in the BOLD signal that is not explained by the model. With $\epsilon_{rv}$ assumed to be normally distributed with mean 0 and estimated variance $\sigma_{r}^{2}$.

Typically, $y_{rv}$ is a weighted average of voxels within a ROI, weighted by the probability of the voxels belonging to the ROI according to a probabilistic atlas.

Although our design matrix represents the timing and type of experimental events, it does not yet account for the temporal dynamics of the BOLD signal.

### 3.1 HRF and convolution

**Fig. 2.**
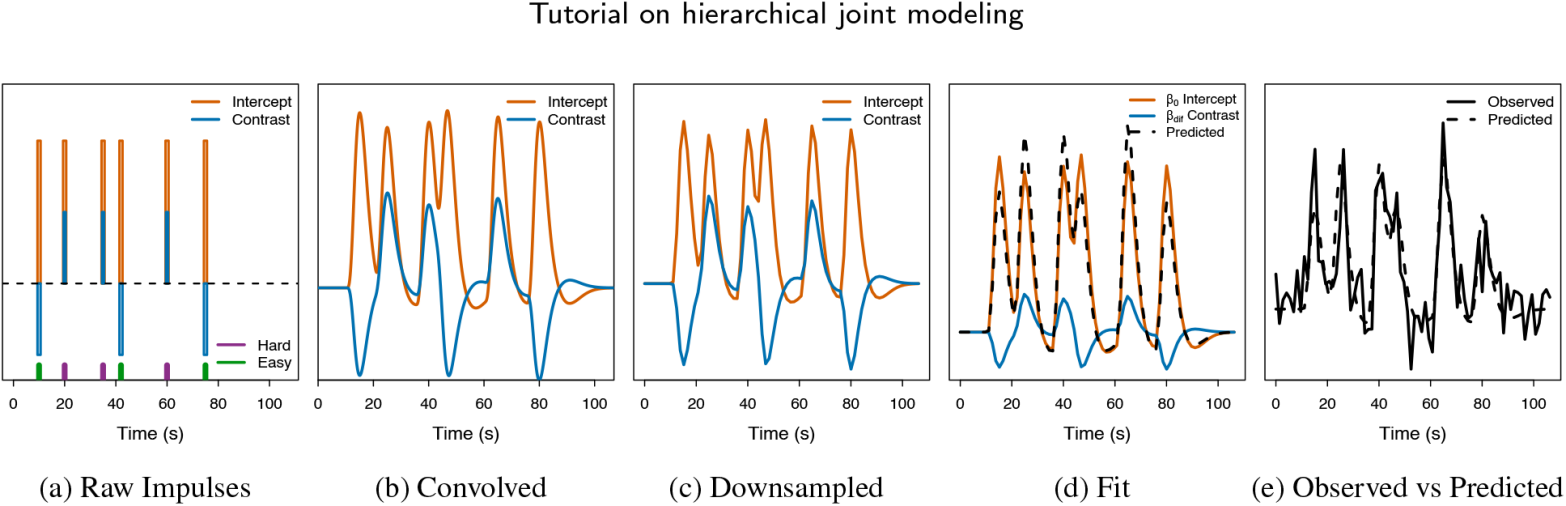
Construction and application of the design matrix for fMRI analysis. (a) The raw impulse design matrix for the experimental design, with the intercept regressor coded as 1 for all events and the contrast regressor coded as 0.5 for Hard and -0.5 for Easy trials. (b) The design matrix convolved with the hemodynamic response function at high temporal resolution. (c) The convolved regressors downsampled to match scanner acquisition times. (d) The downsampled regressors scaled by their respective beta weights obtained from model fitting ($\beta_{0}$ for the intercept and $\beta_{\text{dif}}$ for the contrast) and summed to form the predicted BOLD signal. (e) Comparison of the observed BOLD signal and the predicted BOLD signal.

Whereas the behavioral data consist of a single observation per trial, fMRI data are measured in real time, typically resulting in multiple data points within a single choice trial. To model the fMRI time-series, we use a hemodynamic response function (HRF), which describes the relation between the onset of experimental events (easy or hard trials) and the predicted corresponding BOLD time-series (Logothetis et al., 2001). The design matrix shown in Figure 2a only represents the timing and type of experimental events.

To account for the delayed and temporally extended nature of the hemodynamic response, we convolve the stimulus functions from the design matrix with a model of the HRF (Fig. 2b; Friston et al., 1994). Convolution blends each brief event impulse with the characteristic HRF shape so that each instantaneous spike is spread out over time into a smooth waveform matching the slow rise and fall of the BOLD signal.

The convolution in Figure 2b is computed at a high temporal resolution to accurately represent the continuous hemodynamic response. However, fMRI data are acquired at discrete time points determined by the repetition time (TR) of the scanner, typically on the order of 0.5–3 seconds (Poldrack et al., 2024). Thus, the convolved regressors must be downsampled to match the actual fMRI acquisition times (Fig. 2c). This process ensures that the design matrix used in the GLM has the same number of rows as the number of fMRI volumes acquired during the experiment.

With the downsampled convolved design matrix, we can estimate the regressors (β) that best explain the observed BOLD signal. Figure 2d illustrates how these estimated regressor weights scale the design matrix columns to model the BOLD response. In our example, the intercept regressor is scaled by $\beta_{0}$ (estimated to be 0.5), representing the overall BOLD response magnitude for any trial regardless of condition. The contrast regressor is scaled by $\beta_{\text{dif}}$ (estimated to be 0.2), representing the additional BOLD activation for “Hard” trials compared with “Easy” trials.

This linear combination allows the model to capture both the overall response to trials (through the intercept) and the specific effect of trial difficulty (through the contrast). The total predicted BOLD signal can be computed similarly to Equation (2), which can then be compared with the observed BOLD signal (Fig. 2e).

### 3.2 Filtering and detrending

fMRI time-series are subject to low-frequency drift artifacts that can arise from scanner instabilities, slow head movements, and physiological processes. These low-frequency drifts can obscure the experimental effects and reduce statistical power (Friston et al., 1994). To that end, time-series are often high-pass filtered to remove low-frequency drift artifacts (Worsley et al., 2002).

Another important aspect of fMRI time-series is temporal autocorrelations, caused by a variety of sources including hardware imperfections and physiological and neural mechanisms (Lund et al., 2006). This autocorrelation is typically accounted for with AR(1) models (Olszowy et al., 2019).

Together, these filtering approaches enhance the signal-to-noise ratio and improve the detection of task-related hemodynamic responses (Tanabe et al., 2002).

## 4 Joint Modeling of fMRI and Behavior

In the previous sections, we have described how behavioral data can be modeled using evidence accumulation models (EAMs) and how neural data can be modeled using the general linear model (GLM) with a design matrix. Here we will collectively refer to the EAM parameters for subject i (e.g., drift rate $v_{i}$ and threshold $a_{i}$) as cognitive parameters ${\text{c}}_{i}$ and the GLM parameters for region r as neural parameters $\beta_{ir}$ .

The relatively poor temporal resolution of fMRI data often makes a trial-to-trial link with behavior infeasible. Therefore, fMRI and behavior are typically linked at the participant level (e.g., do people who have higher drift rates also have stronger BOLD responses).

### 4.1 Hierarchical structure

To simultaneously model fMRI and behavior, we can link the cognitive parameters ${\text{c}}_{i}$ to the neural parameters ${\text{n}}_{ir}$ through a joint hierarchical structure:

$$RT_{il},R_{il}=\text{EAM}\left({\text{c}}_{i}\right).$$ (3)

$$y_{iv}=\text{GLM}\left(\beta_{ir}\right).$$ (4)

$$\left[\begin{array}{c} \begin{array}{c} {\text{c}}_{i}\\ \beta_{i1}\\ \vdots \end{array}\\ \beta_{ir} \end{array}\right]∼ℳVN\left(\left[\begin{array}{c} \begin{array}{c} \mu_{c}\\ \mu_{\beta_{1}}\\ \vdots \end{array}\\ \mu_{\beta_{r}} \end{array}\right],\Sigma \right).$$ (5)

Here, $RT_{il}$ and $R_{il}$ represent the two behavioral measurements for participant i on trial l: response time (RT in seconds) and choice response (R, a discrete categorical outcome). These are modeled jointly by the evidence accumulation model (EAM ) governed by the participant-specific cognitive parameter vector ${\text{c}}_{i}$ (which contains parameters such as $v_{0}$ and $v_{\text{dif}}$ ). Similarly, $y_{iv}$ represents the measured continuous BOLD time-series amplitude for participant i in region of interest r during scanner volume v, which is modeled with the general linear model (GLM ) governed by the participant- and region-specific neural parameter vector $\beta_{ir}$ .

At the group level, individual participant parameter vectors (${\text{c}}_{i}$ and all $\beta_{ir}$ ) are assumed to be drawn from a joint Multivariate Normal (ℳVN ) distribution. Here, vector $\mu_{c}$ and vectors $\mu_{\beta_{r}}$ represent the group-level (i.e., across participant) means for the cognitive and neural parameters, respectively. Finally, Σ is the joint covariance matrix capturing both variances and cross-domain correlations between all parameters, detailed in the next section.

### 4.2 The covariance matrix

**Fig. 3.**
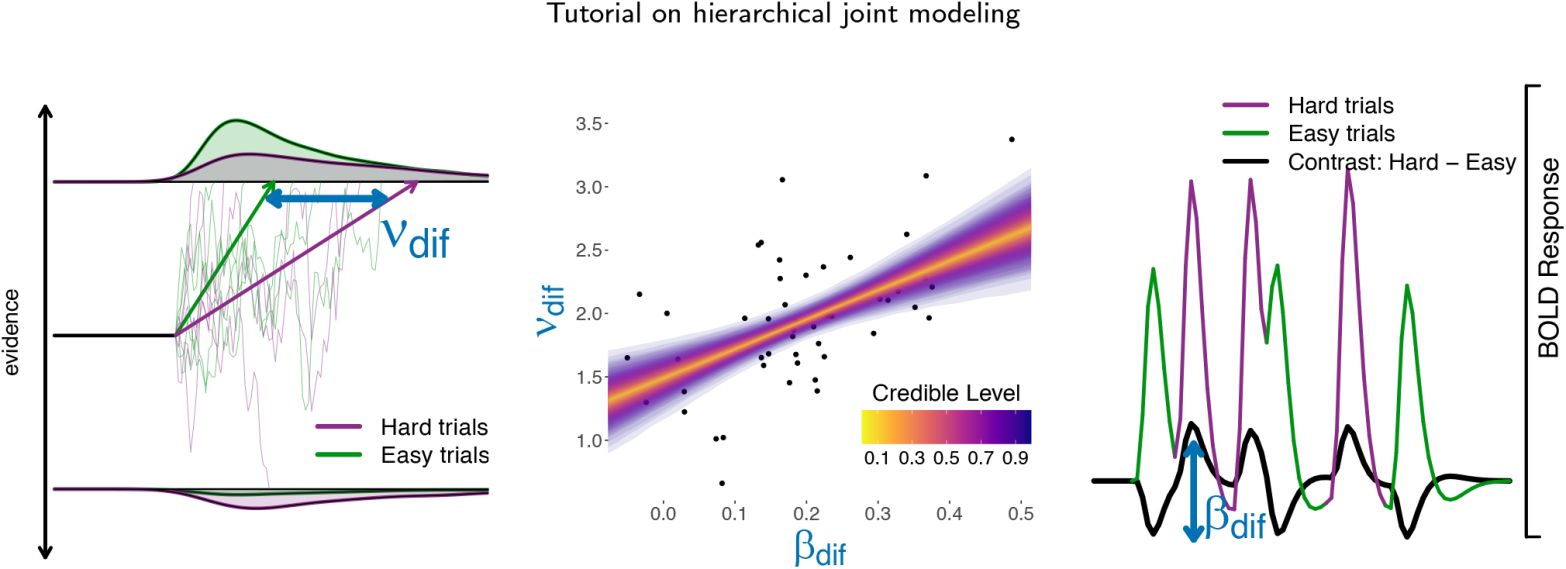
Hierarchical joint model linking neural and behavioral parameters. (left) The diffusion decision model represents the decision-making process, with a drift rate difference parameter ($v_{\text{dif}}$ ) that varies between Easy and Hard conditions. (middle). The joint group-level model captures the correlation between the behavioral parameter $v_{\text{dif}}$ and the neural parameter $\beta_{\text{dif}}$ . The gradient represents the credible interval of the correlation. (right) The neural GLM estimates a difference parameter ($\beta_{\text{dif}}$ ) that scales the regressor for difference in predicted BOLD response between Easy and Hard conditions.

The joint covariance matrix captures the relationships between parameters across participants. For instance, a positive covariance between $v_{\text{dif}}$ and $\beta_{\text{dif}}$ indicates that participants with larger behavioral effects of difficulty also show stronger neural modulation by difficulty (see Fig. 3).

The group-level covariance matrix Σ captures variances and covariances among all cognitive and neural parameters. We partition Σ as

$$\Sigma =\left[\begin{array}{cc} \Sigma_{c} & \Sigma_{\beta ,c}\\ \Sigma_{c,\beta } & \Sigma_{\beta } \end{array}\right],$$ (6)

where

- $\Sigma_{c}$: covariances among cognitive parameters,
- $\Sigma_{\beta }$: covariances among neural parameters across ROIs,
- $\Sigma_{c,\beta }$ : cross-domain covariances that directly encode brain–behavior links.

The covariances are typically standardized to correlations to account for the different scales between the parameters. Rather than estimating all possible correlations, we recommend specifying focused blocks that align with your theoretical brain–behavior hypotheses (see Section 6). This block-wise approach reduces the number of estimated correlations, enhances estimation stability, and keeps inference tightly focused on theoretically meaningful relationships.

In the example illustrated in Figure 3, we link the drift rate difference parameter ($v_{\text{dif}}$ ) from the DDM, which captures the behavioral effect of task difficulty, with the neural contrast parameter ($\beta_{\text{dif}}$ ), which captures the neural effect of task difficulty. The joint distribution (middle panel) shows a positive correlation, suggesting that participants who behaviorally differentiate more between easy and hard trials also show stronger neural differentiation.

### 4.3 Advantages of joint modeling

With these formalisms in mind, we can clarify more specifically what the methodological advances are. First, fitting behavior and fMRI together with a covariance structure (Equation 6) lets information “borrow strength” across domains: weakly identified neural parameters can be stabilized by precise behavioral information, and vice versa. This partial pooling is especially important when the data are noisy or few data points are available.

Second, and more importantly, joint estimation accounts for measurement error. Both behavior (Rouder & Haaf, 2019) and BOLD time-series (Chen et al., 2017, 2021) contain substantial trial-to-trial variability that cannot be accounted for by the model. For example, reaction times may fluctuate due to brief lapses of attention or motor execution noise. BOLD responses are also affected by lapses of attention, but can also be distorted by physiological artifacts (e.g., cardiac and respiratory cycles) or motion.

When cognitive and neural parameters are first estimated separately and correlated in a second step, each estimate is implicitly treated as error-free. By not “filtering” out the measurement error, the resulting correlations are biased toward zero (see Appendix A and Fig. 12) and their certainty intervals are too narrow (Gelman & Hill, 2007; Matzke et al., 2017; Spearman, 1904).

The hierarchical covariance structure of a joint model explicitly accounts for both measurement error and between-participant variability. As a consequence, posterior correlations between brain and behavior are unbiased (recover the true correlation even in the presence of noise), and are properly uncertain, because credible intervals incorporate both sampling error and parameter-estimation error. These properties make joint models the statistically principled choice whenever the scientific question hinges on individual differences in brain–behavior relationships.

## 5 Workflow

**Fig. 4.**
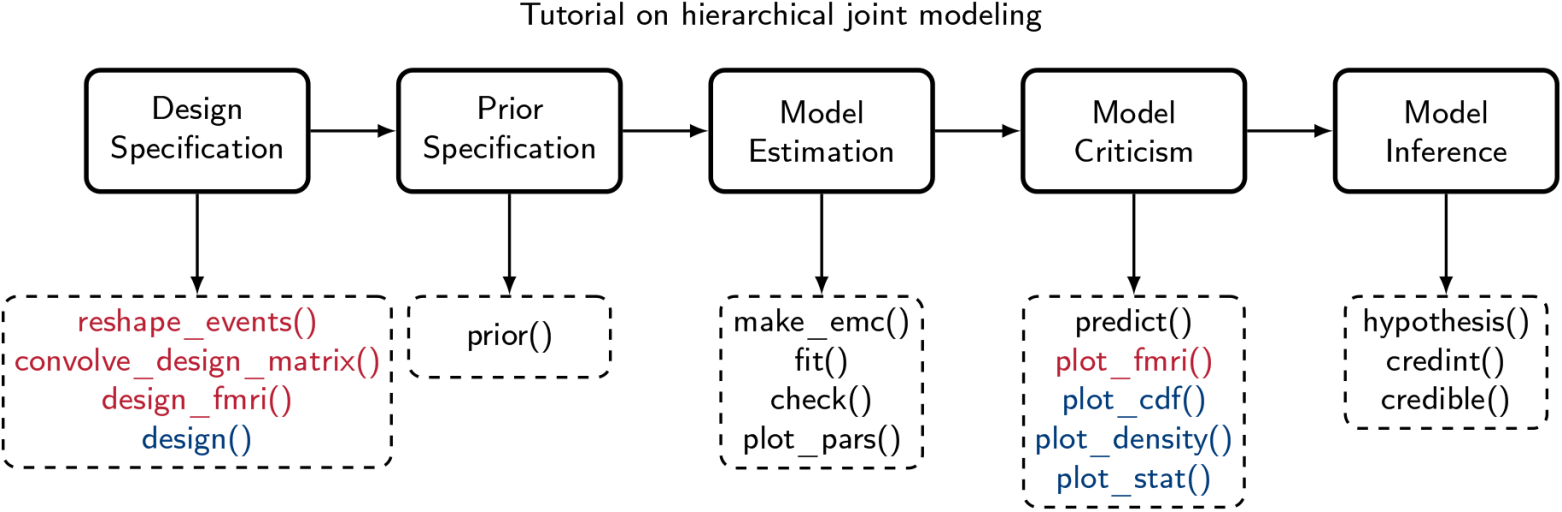
Workflow diagram showing the main steps in the joint modeling process and their associated functions. The functions in red are used for the neural data, the functions in blue are used for the behavioral data, the functions in black are used for both.

The key steps and associated functions used in the joint modeling workflow are outlined in Figure 4. The following section provides a theoretical overview of the workflow, the application section below will further illustrate the implementation of the workflow with code using an example analysis.

The current pipeline in **EMC2** is designed for statistical analysis using Bayesian hierarchical methods, in particular for joint models (though not exclusively). It assumes that the data have already been preprocessed and are available in a dataframe format. Although many different preprocessing pipelines exist, a common, standardized pipeline is offered by fMRIprep (Esteban et al., 2019, 2020), which performs *minimal preprocessing*, which includes motion correction, field unwarping, coregistration to anatomical data, and potentially slice time correction (see Appendix D for full details). If R is preferred for preprocessing, see https://cran.r-project.org/web/views/MedicalImaging.html and Polzehl and Tabelow (2019).

Here, we focus on modeling time-series on regions of interest (ROIs). Additionally, the time-series of each run should be on the same scale. Various options for scaling exist, but here we calculated the percent signal change, using $\left(x-\bar{x}\right)/\bar{x}\times 100$ , where x is the BOLD signal and $\bar{x}$ is the mean BOLD signal within a run.

### 5.1 Installation and data organization

To install the package, run install.packages(“EMC2”) in R. The **EMC2** source code can be found at: https://github.com/ampl-psych/EMC2. The **EMC2** package expects the neural time-series to be organized in a dataframe, with a column for subjects (participant identifier), run, time, and one column per ROI. Here, time refers to the time points at which each volume in the fMRI data was acquired, spaced by the repetition time (TR). The run column codes the different runs in which fMRI data are typically acquired. Similarly, **EMC2** expects the behavioral data organized in a dataframe with columns for subjects, response (R) and the response time (RT), and additional columns for the experimental conditions. With the preprocessed neural and behavioral data, users proceed through the following steps (see also Fig. 4):

### 5.2 Design specification

First we create an events matrix for the neural data. **EMC2** expects the event files in a long format, but wide format events can be converted using the reshape_events function.

The events matrix indicates the timing of the different experimental events (e.g., trial-onset), as well as their duration and modulation. The duration specifies how long (in seconds) we expect the neural activity associated with each event to persist, assuming a simple on–off activation pattern (before considering the hemodynamic response, see Fig. 2a). The modulation specifies the scale of the event. For numeric variables, the modulation is set to their value (unless otherwise specified), and for factor variables, the modulation is set to 1 by default.

Next, the events matrix is convolved with the hemodynamic response function (HRF) using the convolve_design_matrix function (heavily based on the **Nilearn *Python*** package: Abraham et al., 2014), which takes the following key arguments:

- events: The events matrix created in the previous step.
- timeseries: The neural time-series (used to extract the volume times).
- hrf_model: Specifies the HRF model. **EMC2** currently supports the SPM (“spm”; Friston et al., 1994) and Glover (“glover”; Glover, 1999) double-gamma HRF models, with optional temporal derivatives to account for onset deviations (“spm + derivatives”, “glover + derivatives”; Friston et al., 1998).
- covariates: Continuous variables to be added as additional columns in the design matrix.
- factors: Specifies which event types should be modeled under a common factor. In our example experiment, we have a difficulty factor with two levels. factors = list(“difficulty” = c(“Hard”, “Easy”)).
- contrasts: Defines contrasts for factors. The design matrix of the example experiment uses: contrasts = list(“difficulty” = c(1/2, -1/2)).
- high_pass_model: Chooses between cosine basis functions (default, “cosine”) or polynomial basis functions (“poly”) to filter out low-frequency drifts.
- high_pass: Controls high-pass filtering of the design matrix. If high-pass filtering is applied to the time-series, it should also be applied here. Setting TRUE (default) filters the design matrix, FALSE disables filtering. Alternatively, “add” adds the high-pass regressors to the design matrix, in which case the neural time-series do not need to be high-passed prior to model fitting. Note that this will drastically increase the number of estimated regressors, making the model more complex and hard to estimate. If still desirable, we recommend using high_pass_model = “poly”, which will add fewer nuisance regressors, at the cost of potentially less accurate high-pass filtering.

Design matrices are convolved separately for each run, then concatenated across runs for each participant. **EMC2** scales all design columns by the maximum amplitude of the HRF to ensure consistent scaling, which aids estimation and prior specification. This scaling can be turned off by setting scale = FALSE, which preserves the original scale. Users can choose to high-pass filter the time-series data using high_pass_filter function. Last step is to specify the GLM type in the design_fmri function: “MRI” or “MRI_AR1” (accounting for autocorrelation; default).

As with the neural data, we also construct the design matrix for the behavioral data. Users can use the design function to specify how experimental manipulations map onto the pa rameters of the evidence accumulation model (see Section 6). For a detailed overview of the modeling steps of the behavioral data, we refer readers to Stevenson, Donzallaz, et al. (2024) and Section 6 below.

### 5.3 Prior specification

Since we are using Bayesian inference, we need to specify prior distributions for the parameters. By default, **EMC2** uses a normal prior distribution with a mean of 0 and a standard deviation of 1 for the group-level mean parameters (μ). Default priors can be adjusted using the prior function. For the group-level covariance matrix (Σ), default settings lead to uniform priors on the correlations (see Appendix C).

### 5.4 Model estimation

Estimating the joint model is straightforward. The make_emc function takes the prior, the behavioral and neural data, and the behavioral and neural design as input and returns a joint model emc object. Furthermore, users can set which behavioral and neural parameters should be modeled as correlated between participants using the par_groups argument (see Section 6). We strongly advise against using a full covariance matrix (i.e. estimating all possible correlations), since the number of estimated correlations grows quadratically with the number of parameters, which quickly become unfeasible to estimate.

Then using the fit function, the parameters are estimated with Markov Chain Monte Carlo (MCMC) sampling (for an introduction to MCMC estimation, see van Ravenzwaaij et al., 2018). The estimation procedure is based on Gunawan et al. (2020) and further developed in Stevenson, Donzallaz, et al. (2024).

### 5.5 Model criticism

The quality of fit is assessed by comparing the observed data to model predictions that are generated using the posterior distribution of the parameters (predict). These predictions can be compared with the observed behavioral (plot_cdf) and neural (plot_fmri) data.

### 5.6 Model inference

Given an adequate fit (Wilson & Collins, 2019), users can proceed to the second stage of model assessment, model inference. In joint models, typically two types of inference are of interest: *group-mean inference* and *individual differences*.

#### 5.6.1 Group-mean inference

First researchers can engage in the typical tests of the effect of the experimental manipulation on the behavioral and neural parameters separately across participants. These tests target the group-level mean (μ) of a within-participant effect. For example, do participants show lower drift rates or stronger BOLD responses for “Hard” compared with “Easy” trials? These hypothesis tests can be performed using the hypothesis function, which returns the Bayes factor (Kass & Raftery, 1995) for the alternative hypothesis against the null hypothesis.

#### 5.6.2 Individual differences

Second, and often more of interest in the context of joint models, researchers can investigate individual differences in the effect of experimental manipulations. These tests target the variation between participants (captured by the covariance matrix Σ). For example, do participants who show stronger behavioral modulation across difficulty levels also exhibit corresponding neural activity changes? Unfortunately, the hypothesis function is not appropriate for tests on the correlations (see Section 7.2). Instead, we can use the credible function to get the posterior distribution of the correlations between the cognitive and neural parameters.

## 6 Application

Here we provide a practical demonstration on how to use the **EMC2** package to jointly model fMRI and behavior. The code snippets here provide an overview of the different steps involved in the modeling process. Some of the processing steps are not detailed here for brevity. To gain a detailed understanding of the different steps, we encourage the reader to work along with the accompanying R code, which can be found at https://osf.io/jxhcp/.

We reanalyze data from Pereira et al. (2020) (data extracted from https://openneuro.org/datasets/ds002158/versions/1.0.2). In this study, participants performed a perceptual decision-making task where they had to determine the direction of motion in a random dot kinematogram (RDK; Britten et al., 1992). The difficulty of the task was manipulated by varying the coherence of the dots (i.e., the number of dots moving in the same direction). In contrast to the example experiment, difficulty was manipulated on a continuous scale (rather than just “Hard” and “Easy” trials).

After each response, participants also provided a confidence rating, indicating how certain they were about their decision. This experimental design allows us to investigate the effect of task difficulty and confidence on neural activity and behavior.

### 6.1 Creating the design matrix

We start by reshaping the events matrix to the correct format. Currently, the events matrix is in wide format, with one column for each experimental condition:

head(events_wide)

**Table:** 

subjects	run	onset	intercept	rt	confidence	difficulty
2	1	4.44	1	0.58	0.11	0.04
2	1	14.36	1	0.66	-0.01	0.21
2	1	24.20	1	0.40	-0.79	0.21
2	1	33.36	1	0.50	-0.02	0.39
2	1	43.24	1	0.56	-0.15	0.39
2	1	52.36	1	0.50	-0.06	0.21

The confidence column refers to the confidence rating, and difficulty to the difference in dots coherence. We demeaned the difficulty and confidence variables and scaled them to by their maximum value. The intercept column is set to 1 for all trials, to add an intercept to the design matrix that captures the overall BOLD response to any trial regardless of condition.

We use the reshape_events function to convert the events matrix to the long format, also setting the duration and modulation for each event type:

events <- reshape_events(events_wide,

    event_types = c(“intercept”, “difficulty”, “confidence”, “rt”),

     duration = list(rt = function(x) x$rt),

      modulation = list(rt = 1))

head(events)

**Table:** 

subjects	run	Onset	modulation	event_type	duration
2.00	1.00	4.44	1.0000	intercept	0.001
2.00	1.00	4.44	0.0382	difficulty	0.001
2.00	1.00	4.44	0.1099	confidence	0.001
2.00	1.00	4.44	1.0000	rt	0.580
2.00	1.00	14.36	1.0000	intercept	0.001
2.00	1.00	14.36	0.2137	difficulty	0.001

Here we follow Mumford et al. (2024) and include RT in the design matrix with constant modulation, and a duration set to the observed RT of that trial. This approach reduces RT-induced confounds and ensures that differences in activation more accurately reflect genuine cognitive effects rather than mere differences in response time.

### 6.2 Convolving with the HRF

Next, we use the convolve_design_matrix function, the most important part of the modeling of the BOLD response (Fig. 2).

design_matrix <- convolve_design_matrix(timeseries, events, covariates = c(“difficulty”, “confidence”), factors = list(“intercept” = “intercept”, “rt” = “rt”), hrf_model = “spm”, high_pass = TRUE, high_pass_model = “cosine”)

Both difficulty and confidence are specified as covariates, since they are continuous variables. In contrast to the difficulty and confidence columns, the intercept and RT columns are intercept parameters (RT modulation is 1, only duration varies) and are, therefore, set as factors (although with just one level).

We further specify that we want to use the spm HRF model. And since we also high-pass filtered our time-series (using high_pass_filter(timeseries)), we set high_pass = TRUE to also filter the design matrix using cosine basis functions (default). The resulting convolved design matrix can be used in the design_fmri function that defines the GLM type: AR(1) (MRI_AR1) or the conventional GLM (MRI) and constructs a “design” object that can be used with **EMC2** functionality:

design_MRI <- design_fmri(design_matrix, model = MRI_AR1)

**Fig. 5.**
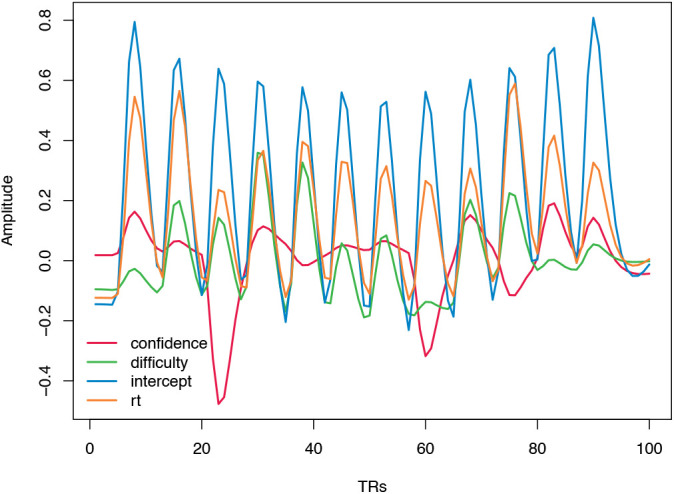
Convolved design matrix for the four event types of interest. The peaks of the intercept BOLD response vary with time due to the high-pass filtering of the design matrix.

The convolved design matrix can also be visualized using the plot_design_fmri function, to showcase the different event types and their associated design matrix columns (Fig. 5). By default, the first 100 TRs are plotted.

plot_design_fmri(design_MRI)

### 6.3 Behavioral modeling

For the behavioral side of the joint model, we again use the DDM to model the evidence accumulation process (Ratcliff, 1978; Ratcliff et al., 2004). The design function maps experimental manipulations to DDM parameters. **EMC2** maps the levels of the response (R) factor of the data to the upper (“Right”) and lower (“Left”) boundary of the DDM. Consequently, we expect negative drift rates (toward the lower boundary) for left-moving stimuli (S) and positive drift rates for right-moving stimuli (6). We formalize this expectation using the contrasts argument. The behavioral data are stored in the behavior data frame. For illustrative purposes, we first create a simple design without the difficulty and confidence mapping:

design_behav <- design(data = behavior, formula = list(v ~ S, a ~ 1, t0 ~ 1, Z ~ 1, sv ~ 1), contrasts = list(S = cbind(d = c(-1, 1))), model = DDM, constants = c (v = 0))

In addition to the drift rates, we further estimate boundary separation (a), non-decision time (t0), starting point bias (Z), and between-trial drift rate variability (sv) parameters. To illustrate the mapping of the drift rates to the different experimental conditions, we can visualize the proposed design mapping using the mapped_pars function:

mapped_pars(design_behav)

$v

 S

 left: v - v_Sd

 right: v + v_Sd

Here S stands for stimulus and d for difference in the v_Sd parameter. This shows that we have reparameterized the drift rate into an intercept (v) parameter and a difference parameter (v_Sd). v_Sd corresponds to the traditional drift rate parameter: higher values lead to faster and more accurate responses (for both stimuli). In contrast, v represents drift bias (higher values favor rightward responses, lower values favor leftward responses). Here we chose to capture response bias using the starting point bias parameter (Z), and set drift bias to 0 using the constants argument. Lastly, to visualize the implied drift rate mapping, we can use the plot function, using a vector of plausible parameter values (p_vector; see prior section):

plot(design_behav, p_vector, factors = list(“v” = “S”))

**Fig. 6.**
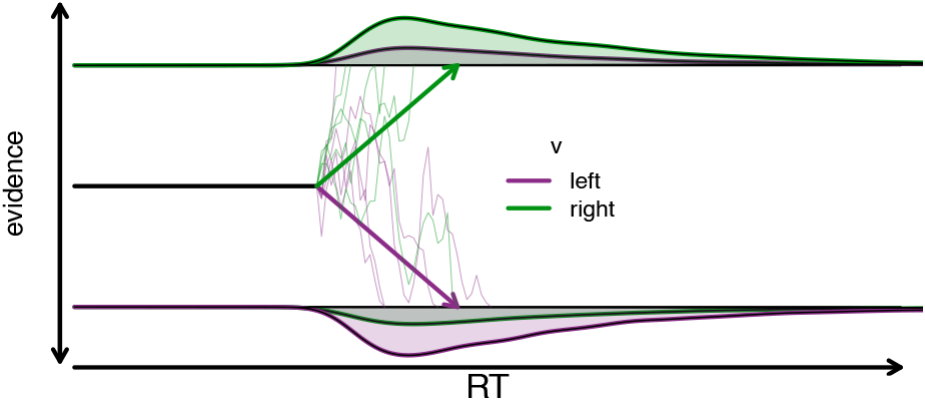
Implied drift rate mapping for left and right moving stimuli.

Figure 6 shows the positive drift rate for right stimuli and negative for left stimuli, resulting in predominantly rightward responses (green) for right moving stimuli and leftward responses (purple) for left moving stimuli. We will now construct our final design, by adding the difficulty and confidence parameters:

design_behav <- design(data = behavior, formula = list(v ~ S(difficulty + confidence), a ~ 1, t0 ~ 1, Z ~ 1, sv ~ 1), model = DDM, contrasts = list(S = cbind(d = c(-1, 1))), constants = c(v = 0, v_difficulty = 0, v_confidence = 0))

Sampled Parameters:

“v_Sd” “v_Sd:difficulty” “v_Sd:confidence” “a” “t0” “Z” “sv”

This new design models the drift rate (v_Sd) as a linear combination of the difficulty and confidence parameters, with v_Sd:difficulty and v_Sd:confidence as the corresponding weights. We further set the drift bias, as well as the modulation weights for the drift bias to 0 using the constants argument.

### 6.4 Prior specification

With the behavioral and neural design matrices constructed, we can now specify the prior distributions for the parameters of interest. We first specify which ROIs to include in the model. Here we follow Pereira et al. (2020) and include left and right inferior frontal gyrus (IFG.l, IFG.r), left and right caudate nucleus (cau.l, cau.r), left and right insula (ins.l, ins.r), left and right nucleus accumbens (nacc.l, nacc.r), and left and right putamen (put.l, put.r). The joint design and joint data are constructed as such:

joint_data <- c(list(behavior), timeseries)

joint_design <- c(list(design_behav), rep(list(design_MRI), length(timeseries)))

names(joint_design) <- c(“beh”, names(timeseries))

This creates a design list with the behavioral design matrix as the first element, and the neural design matrices as the remaining elements (one per ROI). Each element of the neural design list is named after the corresponding time-series, and the behavioral design matrix is named “beh”. This ensures that all parameter names are unique and that the parameter names are consistent across the design list.

This design list is used to specify the prior distributions for the parameters of interest, and subsequently fit the model. Here we only specify the prior distributions for the group-level means (μ; mu in **EMC2**), the prior settings for the variance-covariance matrix are left to default (see Appendix C). We use the sampled_pars function to get the parameter names that are sampled from the design matrix. We start by setting the following priors for the behavioral parameters:

prior_beh <- sampled_pars(design_behav)

prior_beh <- c(1, 0, 0, log(1), log(0.35), qnorm(.5), log(.3))

This yields the following vector of parameters:

v_Sd v_Sd:difficulty v_Sd:confidence    a

1    0    0    log(1)

t0   Z   sv

log(0.35) qnorm(.5) log(.3)

Notice the transformations (log, qnorm). **EMC2** transforms parameters that are constrained to be positive using a log transform (e.g., non-decision time or response boundary separation) and parameters that are constrained to be between 0 and 1 (e.g., relative starting point bias) using a probit transform (qnorm). These transformations ensure that the parameters have support on the real line (i.e., [-∞, ∞]). This is important for joint models, since correlations assume that the parameters are normally distributed and that their values are not restricted in range. When specifying priors, we have to take these transformations into account. To see all transformations, see ?DDM and ?MRI_AR1. We now assign the behavioral priors to the group-level means:

prior_mu <- sampled_pars(joint_designprior_mu[grepl(“beh”, names(prior_mu))] <- prior_beh

Here the grepl function is used to select the parameters that contain the string “beh” in their names. For the neural parameters, we have no a priori expectations for the regression coefficients, so we leave the default settings (normal prior with mean 0 and variance 1). In addition to the regression coefficients, the MRI_AR1 model also estimates the residual standard deviation (sd; log-transformed) and an AR1 autocorrelation parameter (rho; probit-transformed) for each ROI. For these we set the following priors:

prior_mu[grepl(“sd”, names(prior_mu))] <- log(.5)

prior_mu[grepl(“rho”, names(prior_mu))] <- qnorm(.2)

Here we use grepl to select the parameters that contain the string “sd” or “rho”, saving the work of specifying the indices for all 10 ROIs manually. We then construct the prior using the prior function:

joint_prior <- prior(joint_design, mu_mean = prior_mu)

To ensure our prior specifications are consistent with our prior beliefs, we plot the prior distributions for the group-level means:

plot(joint_prior)

**Fig. 7.**
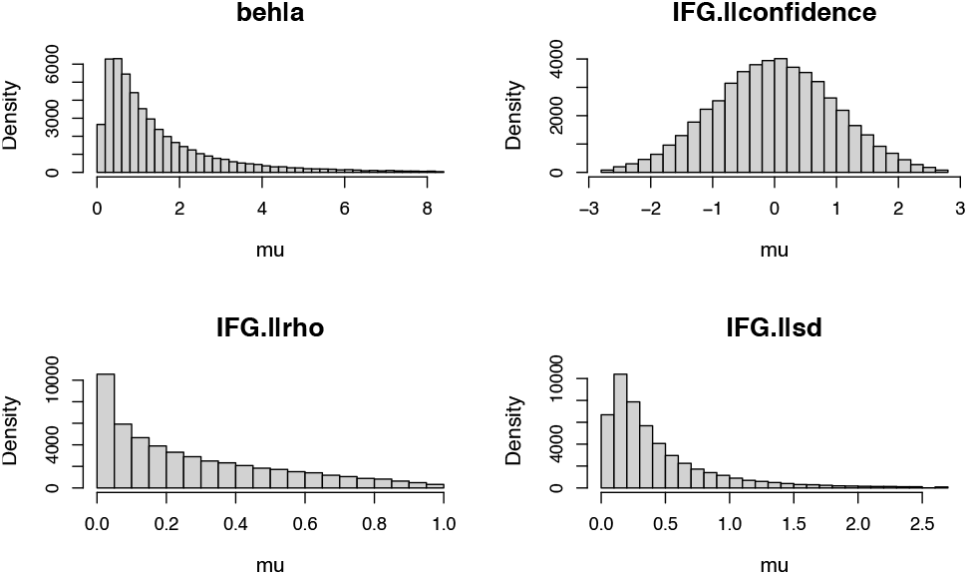
Priors for the boundary separation (beh|a), and for the left IFG the confidence regressor (IFG.l|confidence), the autocorrelation parameter (IFG.l|rho), and the residual variance (IFG.l|sd).

Figure 7 shows the priors for the boundary separation, the confidence regressor, the autocorrelation parameter, and the residual variance for the left IFG. The prior plots for the other parameters are omitted for brevity.

### 6.5 Model estimation

With the set-up complete, we can now fit the model. We first create a joint model object using the make_emc function. Here we use a blocked covariance matrix, since estimating correlations between all 77 parameters of the current joint model would result in 2926 unique correlations! To define our blocked covariance matrix, we again use the grepl function to create three parameter groups:

par_names <- names(sampled_pars(joint_design)) confidence <- par_names[grepl(“confidence”, par_names)] difficulty <- par_names[grepl(“difficulty”, par_names)] intercept <- c(par_names[grepl(“intercept”, par_names)], “beh|v_Sd”)

These parameter groups are used to test the hypotheses that the difficulty and confidence-related modulation of the BOLD response correlate with its behavioral impact on decision making. The correlations between the intercept drift rate and the intercept (trial-onset) BOLD response are included to filter out the covariance associated with attention and arousal effects of trial-onset and the drift rate.

This grouping structure focuses our analysis on theoretically meaningful brain–behavior relationships and keeps the model computationally tractable. We now use the make_emc function, passing the joint data, design, parameter groups, and prior to create the joint model object:

joint_model <- make_emc(joint_data, joint_design, par_groups = list(intercept, confidence, difficulty), prior_list = joint_prior)

We can then pass the joint model object to the fit function to fit the model:

joint_model <- fit(joint_model, cores_per_chain = 3, fileName = “joint_model.RData”)

The fileName argument is used for intermediate storage of the model. By default, **EMC2** uses three chains and parallelizes across these chains, specifying cores_per_chain = 3, thus uses nine cores (3 chains × 3 cores per chain).

**Fig. 8.**
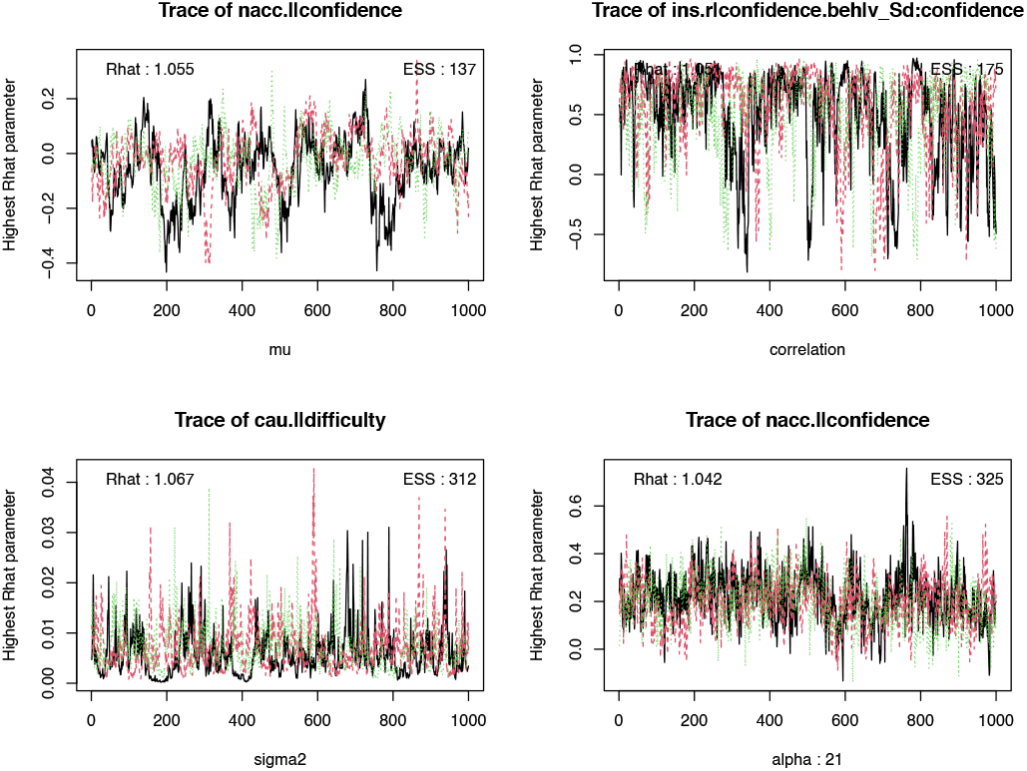
Trace plots of the parameters of the worst converged parameter of the group-level means (mu), standard deviation (sigma2), correlation (correlation), and subject-level parameters (alpha). The chains show good convergence.

Estimation took 50 minutes on a Macbook M4 Pro. Bayesian parameter estimation is computationally and memory intensive, especially since we are estimating a 1870 parameter hierarchical model. **EMC2** is highly optimized for Bayesian hierarchical estimation. After estimation, we use the check function (Fig. 8) to check the convergence of the chains:

check(joint_model, selection = c(“mu”, “correlation”, “sigma2”, “alpha”))

Here mu, correlation, sigma2, and alpha represent the group-level means, correlations, standard deviation, and subject-level parameters, respectively. The check function displays MCMC plots for the worst converged parameter in each group. The x-axis represents the iteration number, the y-axis represents the parameter value, and the color represents the chain.

The plots also show the $\hat{R}$ (Rhat) statistic (values near 1 indicate good convergence; Gelman & Rubin, 1992), and effective sample size (ESS), which accounts for autocorrelation in the MCMC chains. The chains show good convergence, although the ESS for the group-level means and correlations is low, which might warrant longer estimation.

### 6.6 Model criticism

Given the convergence of the chains, we can check the fit of the model by comparing the observed data to model predicted data, using the predict function:

post_predict <- predict(joint_model)

This returns a named list of posterior predictive datasets (50 replicates by default) for both behavioral and ROI-specific neural data, which we use to evaluate fit quality and uncertainty across the posterior predictives.

To plot the observed and predicted data, we can use the plot_fmri function. For example, here we plot the predicted BOLD response for the right inferior frontal gyrus (IFG.r) for the intercept event type (i.e., the mean BOLD response to a trial):

plot_fmri(joint_data$IFG.r, post_predict$IFG.r, event_type = “intercept”, events = events)

The plot_fmri function visualizes event-related changes in brain activity (BOLD signal) using a finite impulse response (FIR) approach. To assess model fit, FIR is applied to both the observed and posterior predictive data generated from the joint HRF model separately. FIR is a more flexible, and almost model-agnostic approach that treats each TR as its own predictor and thus assumes no fixed shape of the BOLD response. For every event occurrence, the signal is modeled from 2 seconds before to 18 seconds after. The solid line is the average response (black = data; green = predictives) across all events, demeaned based on the pre-event weights. The shaded area shows where 95% of the model’s predictive curves fall, giving a sense of uncertainty. Because FIR provides a relatively flexible characterization of the BOLD response, discrepancies between the observed and model-predicted FIR curves can be interpreted as potential HRF model misfit.

**Fig. 9.**
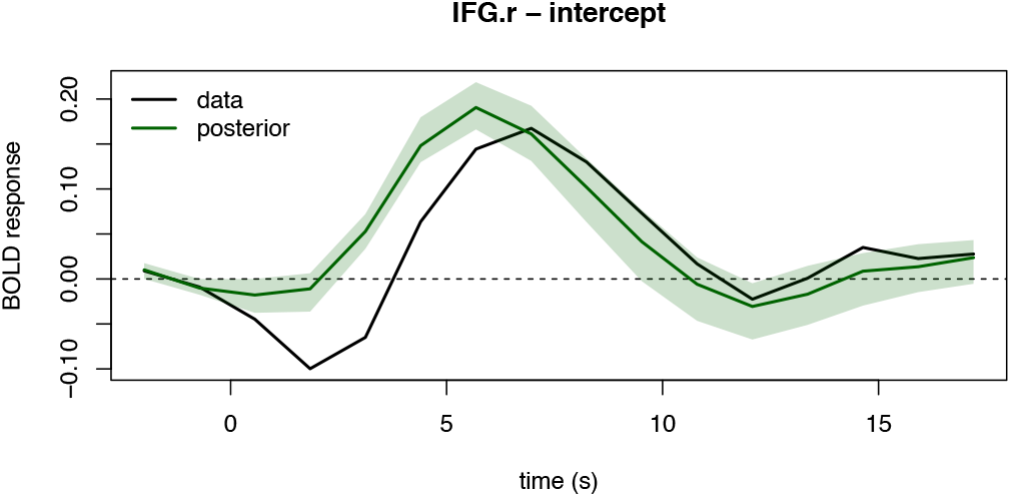
Observed (black) and predicted (green) right inferior frontal gyrus (IFG.r) BOLD response modulation by intercept (i.e., the mean BOLD activation on trial-onset). The shaded area represents the 95% credible interval of the predictions.

**Fig. 10.**
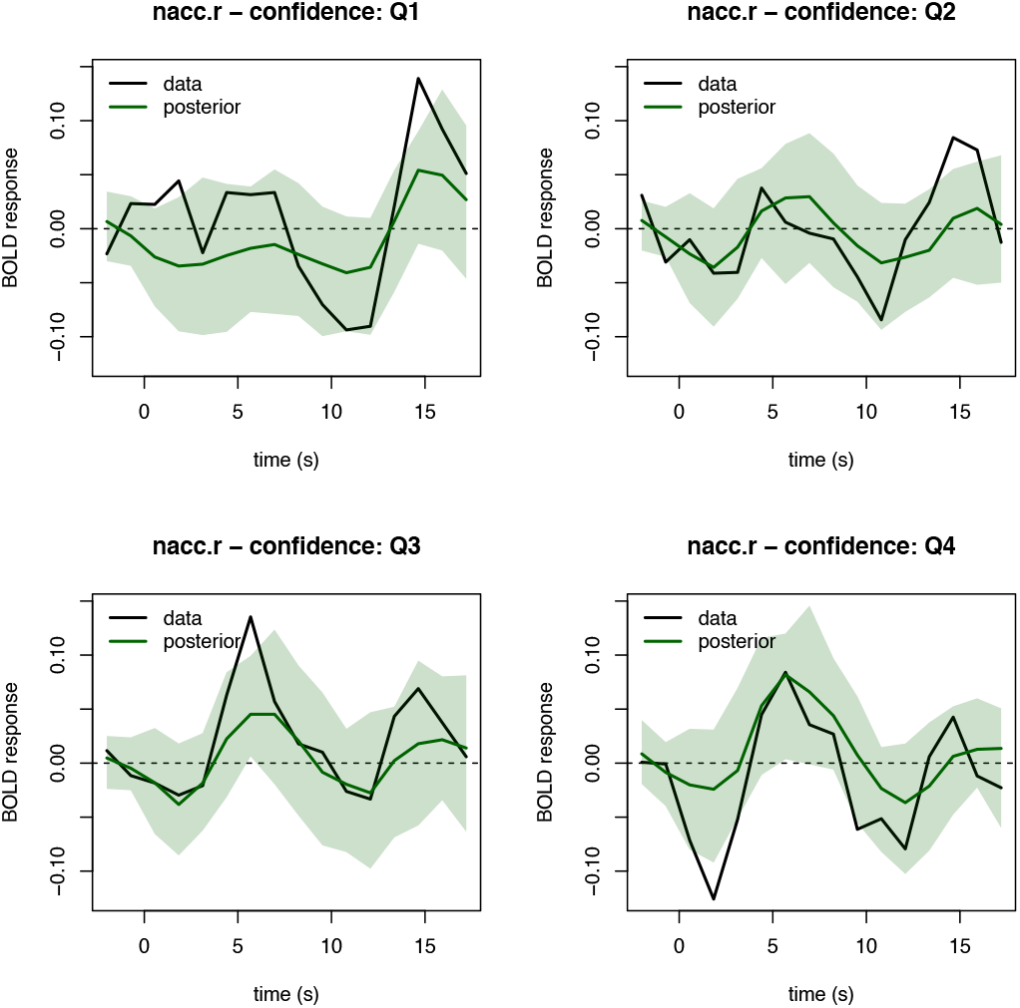
Observed (black) and predicted (green) right nucleus accumbens (nacc.r) BOLD response modulation by confidence divided in four quantiles (Q). The shaded area represents the 95% credible interval of the predictions.

Figure 9 shows that the model captures the shape of the BOLD response, but the response is consistently overestimated, suggesting that the canonical HRF might not fit the BOLD response in the right inferior frontal gyrus. We can also illustrate the BOLD response to a continuous value using the plot_fmri function (Fig. 10):

plot_fmri(data$nacc.r, post_predict$nacc.r, event_type = “confidence”, events = events)

By default, **EMC2** groups continuous values into four quantiles (Q1 to Q4) to facilitate interpretation (Fig. 10). Note that the BOLD response is stronger in trials where participants reported higher confidence. This pattern is well captured by the model. We omit plots of other ROIs for brevity, but they can be made analogously.

**Fig. 11.**
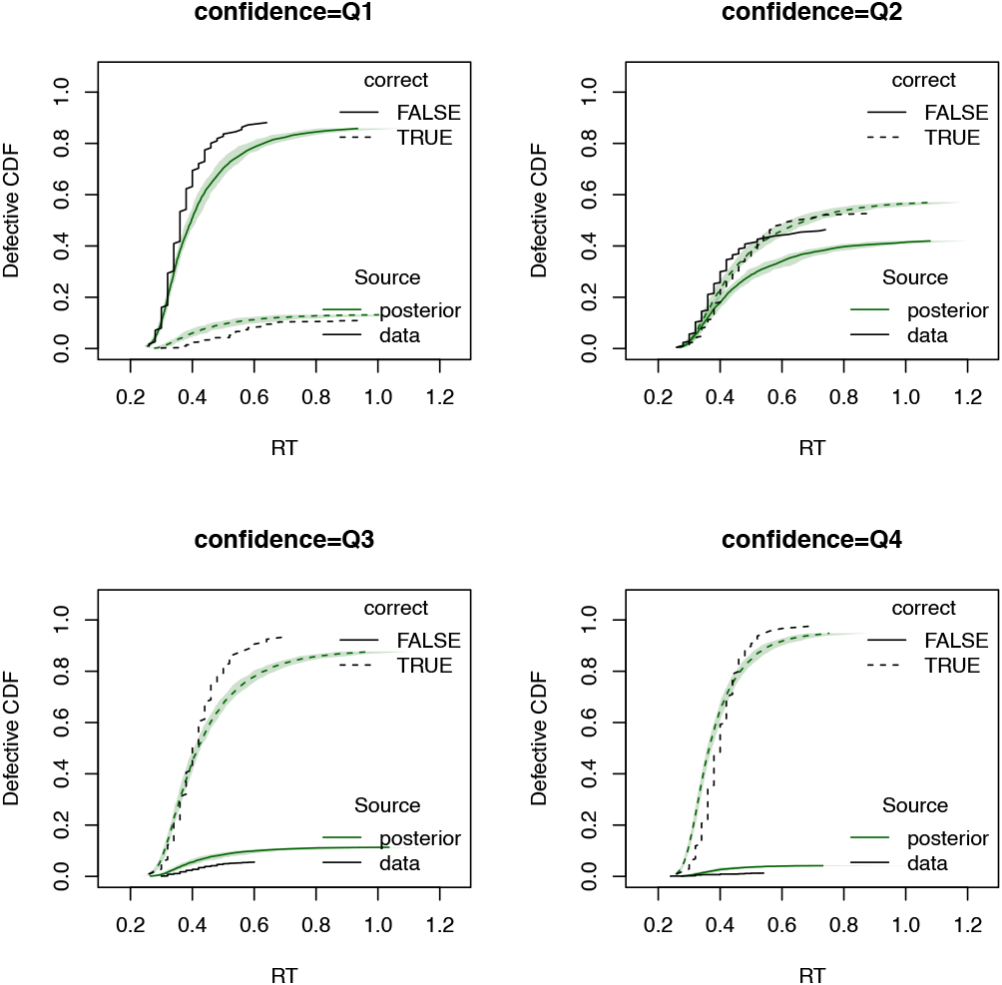
Defective cumulative distributions functions (CDFs), the probability of a response, p(R), as a function of response time (RT) for empirical data (black line) and posterior-predictive data (green lines indicate median across replicates, shaded area indicates 95% credible intervals). The posterior-predictive CDF is calculated by aggregating over replicates.

To compare the predicted and observed behavior, we can use the plot_cdf (or plot_density) function (Fig. 11):

acc_fun <- function(data) return(data$R == data$S) plot_cdf(joint_data$beh, post_predict$beh, defective_factor = “correct”, factors = “confidence”, functions = list(correct = acc_fun))

The x-axis represents the response time, and the y-axis represents the cumulative probability of having made a response by that RT. These plots show that both RTs and the proportion of incorrect responses are overestimated by the model. Also note that people make more errors than correct responses at the lowest confidence levels.

### 6.7 Model inference

Lastly, we perform model inference. We will first test group-level mean effects and then individual differences.

#### 6.7.1 Group-mean inference

We will test whether the experimental manipulations had a group-level effect, by calculating the Bayes factors for the intercept (trial-onset, independent of experimental manipulation), confidence, and difficulty effects on the drift rate and ROI BOLD response regressors, using the hypothesis function. Here, for example, we calculate the Bayes factor for the hypothesis that across participants the right insula (ins.r) BOLD response is modulated by confidence against the null hypothesis, using the group-level mean of the parameter.

hypothesis(joint_model, parameter = “ins.r|confidence”) 0.39

We repeated this for all group-level mean parameters of interest. The results are shown in Table 1. There is moderate evidence that the drift rate is modulated by confidence and difficulty. Most ROIs show increased BOLD responses to a trial event. In contrast, only the left IFG is convincingly modulated by confidence.

**Table 1.** Bayes factors for intercept (trial-onset), confidence, and difficulty across the drift rate and ROIs.

	Intercept		Confidence		Difficulty	
Drift rate	> 1000		2.67		6.57	
	Left	Right	Left	Right	Left	Right
IFG	0.837	0.067	2.95	0.568	0.094	0.076
Caudate	1.39	0.411	0.241	0.119	0.025	0.035
Insula	> 1000	> 1000	0.536	0.384	0.025	0.024
NAcc	7.63	0.448	0.089	0.071	0.056	0.168
Putamen	> 1000	> 1000	0.096	0.092	0.065	0.051

The Bayes factors are calculated against the null hypothesis that the parameter equals 0, using the group-level mean.

#### 6.7.2 Individual differences

Now we engage in the main analysis of interest, to test the brain–behavior relationships across participants. We first note that the absence of a group-level effect does not imply that the brain–behavior relationships are the same across participants. To illustrate, across participants the left and right putamen might not be modulated by confidence; however, it would still be interesting to test whether participants who had higher confidence modulation also showed higher drift rate modulation.

To test the brain–behavior relationships across participants, we use the credible intervals of the posterior distributions of the correlations of interest (unfortunately the hypothesis function is not applicable for correlations see Section 7.2):

credint(joint_model, selection = “correlation”, use_par = c(“beh|v_Sd:difficulty”, “beh|v_Sd:confidence”))$”beh|v_Sd:difficulty”

2.5% 50% 97.5%

IFG.l|difficulty -0.548 0.023 0.568

IFG.r|difficulty -0.520 0.077 0.635

cau.l|difficulty -0.544 0.076 0.654

. . .

$”beh|v_Sd:confidence”

2.5% 50% 97.5%

IFG.l|confidence -0.662 0.407 0.913

. . .

Recall that we specified correlation blocks linking three parameter types: intercept drift rate with intercept (trial-onset) ROI regressors, confidence drift rate with confidence ROI regressors, and difficulty drift rate with difficulty ROI regressors. The credint function returns credible intervals for correlations between these drift rates and their associated ROI regressors. Here we focus on the main correlations of interest, between the confidence modulation of the drift rate and the BOLD response and the difficulty modulation of the drift rate and the BOLD response.

Table 2 highlights that the confidence modulation of the drift rate is positively correlated with the confidence modulation of the BOLD response in the right nucleus accumbens (nacc.r). We found no correlation between the difficulty modulation of the drift rate and the difficulty modulation of the BOLD response in any ROI.

**Table 2.** Posterior correlations (median [2.5 %, 97.5 %]) between drift-rate parameters and neural ROIs, by hemisphere.

	Drift-rate correlations			
	Confidence		Difficulty	
	Right	Left	Right	Left
IFG	0.41 [–0.66, 0.91]	0.41 [–0.66, 0.91]	0.08 [–0.52, 0.64]	0.02 [–0.55, 0.57]
Caudate	0.56 [–0.55, 0.95]	0.61 [–0.47, 0.92]	0.10 [–0.54, 0.66]	0.08 [–0.54, 0.65]
Insula	0.63 [–0.45, 0.92]	0.23 [–0.69, 0.86]	0.09 [–0.48, 0.61]	0.07 [–0.49, 0.62]
NAcc	**0.87** [0.07, 0.97]	0.60 [–0.55, 0.94]	0.10 [–0.54, 0.67]	0.05 [–0.51, 0.60]
Putamen	0.72 [–0.39, 0.94]	0.51 [–0.60, 0.91]	0.11 [–0.53, 0.67]	0.06 [–0.55, 0.64]

Correlations whose 95% credible interval excludes 0 are in bold.

### 6.8 Attenuation

**Fig. 12.**
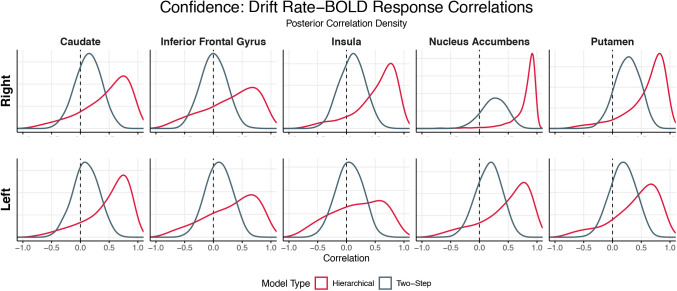
Comparison of the correlation estimates between the confidence modulation of the drift rates and the BOLD response across different brain regions. The two-step approach (gray) shows clear attenuation of correlations (bias toward zero) compared with the hierarchical joint model (red), which properly accounts for measurement error in both behavioral and neural parameters.

To illustrate the advantage of joint modeling, we separately ran a two-step modeling approach, where we first estimated the behavioral and neural parameters separately for each participant, and then correlated the drift rate parameters with the associated BOLD responses (see code for details). The results for the confidence correlations are shown in Figure 12. Note that the two-step approach shows clear attenuation of the correlations (bias toward zero) compared with the hierarchical joint model, which shows higher correlation estimates. Furthermore, the hierarchical approach frequently has wider credible intervals, since the measurement error is properly accounted for and propagated through the model. Figure 12 also highlights that we would have incorrectly made the conclusion that there is no credible correlation between the confidence modulation of the drift rate and the BOLD response in the right nucleus accumbens if we had used the two-step approach.

### 6.9 Application discussion

Here we used **EMC2** to jointly model fMRI and behavior in one joint model. This allows us to test specific hypotheses about the brain–behavior relationship. Our group-level mean analysis showed that the drift rate was modulated by confidence and difficulty, and that all ROIs showed differential BOLD responses in response to a trial, except for the left IFG. We further found that activity in the left and right IFG was modulated by confidence.

Our individual-differences analysis showed positive correlations between drift rates and BOLD responses to a trial in all ROIs. Thus, people who showed stronger neural modulation to trial-onset (potentially due to increased attention) also performed better at the task. More importantly, we found a positive correlation between the effect of confidence on the drift rate and the effect of confidence on the BOLD response in the right nucleus accumbens. This finding aligns with previous work suggesting a relationship between the ventral striatum (which includes the nucleus accumbens) and behavioral measures of confidence (Guggenmos et al., 2016; Schwarze et al., 2013).

## 7 Other Considerations

Before we conclude, we would like to briefly discuss some areas of interest for future research.

### 7.1 Shape of the BOLD response

Typical fMRI applications include temporal derivatives of regressors, to account for deviations from the canonical HRF (Friston et al., 1998). **EMC2** does support the inclusion of temporal derivatives; however, including temporal derivatives also changes the interpretation of the regressor of interest (see Appendix B for details). Furthermore, it drastically increases the number of estimated parameters, and thus the model complexity.

Future work may explore estimating convolution parameters within the model itself, such that the HRF shape can be estimated from the data. This will allow for more parsimonious and accurate BOLD response modeling, as well as improved interpretation.

### 7.2 Hypothesis tests for correlations

Here we made statistical inferences on the correlations using posterior credible intervals. A more statistically sound approach is Bayesian model comparison, which directly evaluates competing models (those with a specific brain–behavior correlation versus those without) and quantifies the evidence for and against these correlations using Bayes factors (Kass & Raftery, 1995). Currently, **EMC2** offers two methods to calculate the Bayes factors: bridge-sampling (compare function; Gronau et al., 2020) and the Savage–Dickey ratio (hypothesis function; Dickey & Lientz, 1970; Wagenmakers et al., 2010). Neither of these methods are appropriate for model comparison on correlations. Bridge sampling requires a null model that selectively turns off individual correlations, which is generally not possible in covariance matrices (Barnard et al., 2000). The Savage–Dickey ratio requires special mathematical properties in the prior distributions that common prior settings for correlation matrices do not satisfy (for more detailed discussion, see Heck, 2019).

### 7.3 Dimensionality

Another issue with a (blocked) covariance matrix is that the number of estimated correlations grows quickly with the number of parameters. For example, correlating a cognitive model parameter with 5 ROIs results in 10 unique correlations, but with 20 ROIs, this grows to 190 unique correlations, thus quickly becoming unfeasible to estimate. Recent work has suggested representing the brain–behavior relationships using a factor analysis model (Kang et al., 2022; Stevenson, Innes, Gronau, et al., 2024, Stevenson, Heathcote, et al. 2024). Not only does this reduce the number of parameters, but it also facilitates the interpretation of the results by decomposing the brain–behavior relationships into meaningful latent factors. For example, we could group the neural difficulty regressors and the difficulty drift rate into a general difficulty factor and test for support for this factor in the data. **EMC2** already supports joint factor analysis models (Stevenson, Heathcote, et al., 2024), but this is beyond the scope of this paper.

### 7.4 Whole-brain modeling

Here we have focused on modeling regions of interest (ROIs). However, commonly researchers also study whole-brain BOLD responses that are modeled using voxel-wise GLMs that are subsequently thresholded to identify brain regions that are affected by experimental manipulations (Nichols & Hayasaka, 2003). Unfortunately, due to memory and estimation demands, voxel-wise GLMs using MCMC chains quickly become unfeasible, since a separate set of regressors needs to be estimated for each voxel, while also accounting for spatial correlations.

Besides memory constraints, brain–behavior relationships cannot be modeled for each voxel separately, since the number of brain–behavior relationships quickly grows unmanageable (even with factor analysis). Therefore, a model-based thresholding procedure that can be applied within the joint modeling framework would have to be developed to enable inference about the brain–behavior relationship in a specific brain region.

## 8 Conclusion

This paper introduces a toolbox to bridge the gap between the theoretical and practical implementations of joint modeling fMRI and behavioral data.

We first provided an overview of the modeling steps involved in the general linear model framework that is typically used to model fMRI data, and how this can be extended to a joint model of fMRI and behavior. We discussed the motivations and advantages of joint modeling, as well as the different steps involved in the modeling process. We then demonstrated how to use the **EMC2** package to jointly model fMRI and behavior in an example analysis (Pereira et al., 2020), highlighting how to assess and make inferences from the model.

We hope that the current toolbox combined with the accompanying tutorial will make it more feasible for cognitive neuroscientists to jointly model fMRI and behavior, and to use the results to test specific hypotheses about the relationship between cognition and brain activity.

## Figures and Tables

**Appendix Fig. A1.**
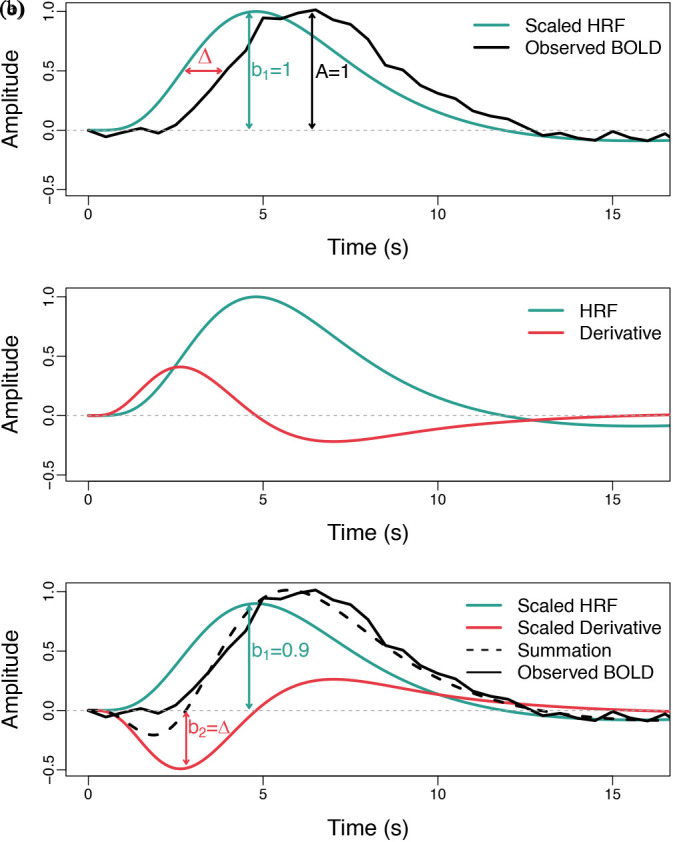
Illustration of using temporal derivatives to account for shifts in the BOLD response. (a) Predicted canonical HRF (green) vs observed BOLD (black) delayed by Δ seconds. The amplitude of the BOLD signal is the same as the canonical HRF. (b) HRF (green) and its temporal derivative (red). The amplitude of the BOLD signal is the same as the canonical HRF. (c) The best-fit HRF (green) and its temporal derivative (red) are combined to predict the observed BOLD signal (black). The amplitude of the BOLD signal is no longer the same as the canonical HRF.

## Data Availability

The source code for the **EMC2** package can be found at: https://github.com/ampl-psych/EMC2. The accompanying tutorial code can be found at: https://osf.io/jxhcp/. The archival data from Pereira et al. (2020) were retrieved from: https://openneuro.org/datasets/ds002158/versions/1.0.2.

## Appendix

### A. Attenuation

A key advantage of joint models is that they explicitly account for measurement error in both the neural and behavioral parameters. When parameters are estimated separately and then correlated (a two-step approach), the resulting correlation estimates are systematically biased toward zero, a phenomenon known as attenuation (Gelman and Hill, 2007; Spearman, 1904).

Consider the correlation between the drift rate parameter from our example DDM ($v_{\text{dif}}$ ) and the corresponding neural parameter ($\beta_{\text{dif}}$ ). When we run a two-step analysis, point estimates (${\hat{v}}_{\text{dif}}$ and ${\hat{\beta }}_{\text{dif}}$ ) are used to calculate the correlation; however, point estimates are contaminated by measurement error:

$${\hat{v}}_{\text{dif}}=v_{\text{dif}}+\epsilon_{v},$$
(7)

$${\hat{\beta }}_{\text{dif}}=\beta_{\text{dif}}+\epsilon_{\beta },$$
(8)

where ${\hat{v}}_{\text{dif}}$ and ${\hat{\beta }}_{\text{dif}}$ are our estimates, and $\epsilon_{v}$ and $\epsilon_{\beta }$ represent measurement errors for the behavioral and neural parameters, respectively. These measurement errors arise from various sources, including trial-to-trial variability, physiological noise, and parameter estimation uncertainty.

Correlations rely on the covariance between the parameters, as well as the variances of the parameters. In the two-step approach, the observed variances are inflated by the measurement error variances:

$$\text{Var}\left({\hat{v}}_{\text{dif}}\right)=\sigma_{v}^{2}+\text{Var}\left(\epsilon_{v}\right).$$
(9)

$$\text{Var}\left({\hat{\beta }}_{\text{dif}}\right)=\sigma_{\beta }^{2}+\text{Var}\left(\epsilon_{\beta }\right).$$
(10)

Although the measurement error variances (Var(ϵ)) cannot always be analytically computed, they can be estimated. This is beyond the scope of this paper, but see Rouder and Haaf (2019) and Donzallaz et al. (2024) for more details. To illustrate the effect of measurement error on the correlation, consider the following true correlation:

$$\rho_{\text{true}}=\text{Corr}\left(v_{\text{dif}},\beta_{\text{dif}}\right)=\frac{\text{Cov}\left(v_{\text{dif}},\beta_{\text{dif}}\right)}{\sqrt{\sigma_{v}^{2}\cdot \sigma_{\beta }^{2}}}.$$
(11)

When we run a two-step analysis, the variance of the point estimates is inflated by the measurement error variances:

$$\rho_{\text{two-step}}=\frac{\text{Cov}\left(v_{\text{dif}},\beta_{\text{dif}}\right)}{\sqrt{\left(\sigma_{v}^{2}+\text{Var}\left(\epsilon_{v}\right)\right)\left(\sigma_{\beta }^{2}+\text{Var}\left(\epsilon_{\beta }\right)\right)}}.$$
(12)

Since the denominator of the correlation in the two-step approach (Equation 12) is larger than the true correlation (Equation 11), the observed correlation will always be attenuated compared with the true correlation. The higher the measurement error relative to the true parameter variability, the larger the degree of attenuation.

This attenuation effect is particularly problematic in neuroimaging studies, where measurement noise can be substantial (Chen et al., 2017). The joint modeling approach addresses this issue by explicitly modeling the measurement process and the hierarchical structure of the data, allowing for estimation of the true correlation between the parameters.

Consequently, instead of the measurement error downward biasing the correlation, the uncertainty in the measurement error is propagated into the posterior distribution of the correlation as demonstrated in Figure 12.

### B. Derivatives

The observed BOLD responses often deviate in onset relative to the canonical HRF (see Appendix Fig. A1a). To account for a small temporal shift Δ, temporal derivatives of the regressors are often included in the design matrix (See Appendix Fig. A1b). This is based on the logic of a first-order Taylor series expansion (Friston et al., 1998). That is, for a small Δ we have

$$\text{HRF}\left(t-\Delta \right)\approx \text{HRF}\left(t\right)-\Delta {\text{HRF}}^{'}\left(t\right).$$

This linear approximation provides an estimate of what the HRF would look like if it were shifted by Δ seconds. Consequently, typical applications of the GLM framework include the temporal derivatives of regressors:

$$y=b_{1}\text{HRF}\left(t\right)+b_{2}{\text{HRF}}^{'}\left(t\right),$$

with $b_{1}$ the regressor of interest and $b_{2}$ the derivative weight that will roughly correspond to -Δ.

However, the inclusion of the derivative term means that the main regressor no longer directly corresponds to the amplitude of the BOLD response (which is usually the quantity of interest). Instead, the amplitude is a function of both $b_{1}$ and $b_{2}$ (Calhoun et al., 2004; Lindquist et al., 2009):

$$A=\text{sign}\left(b_{1}\right)\sqrt{b_{1}^{2}+b_{2}^{2}}.$$

There are different ways to reparameterize the coefficient pair $\left(b_{1},b_{2}\right)$, such that the main regressor corresponds to the amplitude (A) and the other parameter is a nuisance parameter (θ), for example, the polar representation:

$$A=\sqrt{b_{1}^{2}+b_{2}^{2}},\;\theta =\text{arctan}\left(\frac{b_{2}}{b_{1}}\right),$$

so that

$$b_{1}=A\cos\theta \text{ and }b_{2}=A\sin\theta .$$

Although this reparameterization is attractive in that it decouples amplitude (as A) from timing variability (as θ), the amplitude parameter only has positive support, and consequently may break the intended correlation when the original effect is roughly zero centered. Alternatively, we can define the signed amplitude as:

$$A=\text{sign}\left(b_{1}\right)\sqrt{b_{1}^{2}+b_{2}^{2}}.$$

Then, as a measure of the relative contribution of the derivative term:

$$\theta =\frac{b_{2}}{\sqrt{b_{1}^{2}+b_{2}^{2}}},$$

which takes values in the interval [−1,1]. With these definitions, the original coefficients can be recovered by

$$b_{1}=A\sqrt{1-\theta^{2}},b_{2}=A\theta .$$

In this way, A retains the sign information of $b_{1}$, while θ represents the signed proportion of the overall effect that is due to the derivative. However, this reparameterization led to sampling issues due to a sign-switching problem that was likely introduced because the sign of $b_{2}$ is dependent on both the sign of A and θ. Clearly, this is a problem that requires further investigation in future work.

### C. Prior Specification

**EMC2** uses normal priors on the group-level means $\mu ∼N\left(\mu_{0},\Sigma_{0}\right),$ where $\mu_{0}$ and $\Sigma_{0}$ can be specified by the user. By default, $\mu_{0}=0_{p}$ (zero for parameters 1…p ) and $\Sigma_{0}={\text{I}}_{p}$ (a p×p identity matrix). For parts of the group-level covariance matrix Σ that do not include covariances, the prior on the diagonal entries ($\sigma_{1...p}^{2}$) is a mixture of inverse-Gamma distributions that gives rise to a half-t prior distribution on the standard deviations (Gelman, 2006; Wand et al., 2011).

$$\sigma_{p}∼half-t\left(A,v\right),$$
(13)

where A and v are the scale and degrees of freedom of the prior half-t distributions on the standard deviation of parameter p.

Joint models explicitly model the covariance between parameters. For blocks of Σ that include covariances, **EMC2** uses a scale-mixture inverse-Wishart distribution, with mixture weights given by an inverse-Gamma distribution (Huang & Wand, 2013):

$$\begin{array}{c} \Sigma |a_{1}...a_{p}∼\text{Inverse - Wishart}(v+p-1,2v\text{diag}\left(1/a_{1}...1/a_{p}\right)\\ a_{p}∼\text{Inverse-Gamma}\left(1/2,1/A_{p}^{2}\right). \end{array}$$
(14)

This prior has the same half-t distribution on the standard deviations as in the diagonal case described above. Furthermore, this structure allows the prior on the correlations to be separated from the prior on the variances (Huang & Wand, 2013). By default, we set $A_{p}\;=0.3$ and v=2 , which leads to broad and weakly informative priors on the variances and uniform priors on the correlations.

The uniform prior on the correlations is appropriate when we do not have prior information about the brain–behavior relationship, as is common in the cognitive neuroscience literature.

Increasing $A_{p}$ weakens the prior on the variances. Increasing v leads to more zero-centered priors on the correlations, whereas decreasing v leads to more prior support for correlations away from 0 in both directions.

### D. fMRI Preprocessing Details

Results included in this manuscript come from preprocessing performed using fMRIPrep 20.2.6 (Esteban et al. (2019); Esteban et al. (2020); RRID:SCR_016216), which is based on Nipype 1.7.0 (Gorgolewski et al. (2011); RRID:SCR_002502).

#### Anatomical data preprocessing

A total of one T1-weighted (T1w) image was found within the input BIDS dataset. The T1-weighted (T1w) image was corrected for intensity non-uniformity (INU) with N4BiasFieldCorrection (Tustison et al., 2010), distributed with ANTs 2.3.3 (Avants et al., 2008, RRID:SCR_004757), and used as T1w-reference throughout the workflow. The T1w-reference was then skull stripped with a Nipype implementation of the antsBrainExtraction.sh workflow (from ANTs), using OASIS30ANTs as target template. Brain tissue segmentation of cerebrospinal fluid (CSF), white matter (WM), and gray matter (GM) was performed on the brain-extracted T1w using fast (FSL 5.0.9, RRID:SCR_002823, Zhang et al., 2001). Brain surfaces were reconstructed using recon-all (FreeSurfer 6.0.1, RRID:SCR_001847, Dale et al., 1999), and the brain mask estimated previously was refined with a custom variation of the method to reconcile ANTs-derived and FreeSurfer-derived segmentations of the cortical gray matter of Mindboggle (RRID:SCR_002438, Klein et al., 2017). Volume-based spatial normalization to one standard space (MNI152NLin2009cAsym) was performed through nonlinear registration with antsRegistration (ANTs 2.3.3), using brain-extracted versions of both T1w reference and the T1w template. The following template was selected for spatial normalization: ICBM 152 Nonlinear Asymmetrical template version 2009c [Mazziotta et al. (1995), RRID:SCR_008796; TemplateFlow ID: MNI152NLin2009cAsym].

#### Functional data preprocessing

For each of the seven BOLD runs found per subject (across all tasks and sessions), the following preprocessing was performed. First, a reference volume and its skull-stripped version were generated using a custom methodology of fMRIPrep. Susceptibility distortion correction (SDC) was omitted. The BOLD reference was then co-registered to the T1w reference using bbregister (FreeSurfer) that implements boundary-based registration (Greve & Fischl, 2009). Co-registration was configured with six degrees of freedom. Head-motion parameters with respect to the BOLD reference (transformation matrices, and six corresponding rotation and translation parameters) are estimated before any spatiotemporal filtering using mcflirt (FSL 5.0.9, Jenkinson et al., 2002). BOLD runs were slice-time corrected to 0.59 seconds (0.5 of slice acquisition range 0 seconds–1.18 seconds) using 3dTshift from AFNI 20160207 (Cox & Hyde, 1997, RRID:SCR_005927). The BOLD time-series (including slice-timing correction when applied) were resampled onto their original, native space by applying the transforms to correct for head motion. These resampled BOLD time-series will be referred to as preprocessed BOLD in original space, or just preprocessed BOLD. Several confounding time-series were calculated based on the preprocessed BOLD: frame-wise displacement (FD), DVARS, and three region-wise global signals. FD was computed using two formulations following Power (absolute sum of relative motions, Power et al. (2014)) and Jenkinson (relative root mean square displacement between affines, Jenkinson et al. (2002)). FD and DVARS are calculated for each functional run, both using their implementations in Nipype (following the definitions by Power et al. (2014)). The three global signals are extracted within the CSF, the WM, and the whole-brain masks. Additionally, a set of physiological regressors were extracted to allow for component-based noise correction (CompCor, Behzadi et al., 2007). Principal components are estimated after high-pass filtering the preprocessed BOLD time-series (using a discrete cosine filter with 128 seconds cutoff) for the two CompCor variants: temporal (tCompCor) and anatomical (aCompCor). tCompCor components are then calculated from the top 2% variable voxels within the brain mask. For aCompCor, three probabilistic masks (CSF, WM, and combined CSF+WM) are generated in anatomical space. The implementation differs from that of Behzadi et al. in that instead of eroding the masks by two pixels on BOLD space, the aCompCor masks are subtracted a mask of pixels that likely contain a volume fraction of GM. This mask is obtained by dilating a GM mask extracted from the FreeSurfer’s aseg segmentation, and it ensures components are not extracted from voxels containing a minimal fraction of GM. Finally, these masks are resampled into BOLD space and binarized by thresholding at 0.99 (as in the original implementation). Components are also calculated separately within the WM and CSF masks. For each CompCor decomposition, the k components with the largest singular values are retained, such that the retained components’ time-series are sufficient to explain 50% of variance across the nuisance mask (CSF, WM, combined, or temporal). The remaining components are dropped from consideration. The head-motion estimates calculated in the correction step were also placed within the corresponding confounds file. The confound time-series derived from head-motion estimates and global signals were expanded with the inclusion of temporal derivatives and quadratic terms for each (Satterthwaite et al., 2013). Frames that exceeded a threshold of 0.5 mm FD or 1.5 standardized DVARS were annotated as motion outliers. All resamplings can be performed with a single interpolation step by composing all the pertinent transformations (i.e., head-motion transform matrices, susceptibility distortion correction when available, and co-registrations to anatomical and output spaces). Gridded (volumetric) resamplings were performed using antsApplyTransforms (ANTs), configured with Lanczos interpolation to minimize the smoothing effects of other kernels (Avants et al., 2008). Non-gridded (surface) resamplings were performed using mri_vol2surf (FreeSurfer). First, a reference volume and its skull-stripped version were generated using a custom methodology of fMRIPrep. Susceptibility distortion correction (SDC) was omitted. The BOLD reference was then co-registered to the T1w reference using bbregister (FreeSurfer) that implements boundary-based registration (Greve & Fischl, 2009). Co-registration was configured with six degrees of freedom. Head-motion parameters with respect to the BOLD reference (transformation matrices, and six corresponding rotation and translation parameters) are estimated before any spatiotemporal filtering using mcflirt (FSL 5.0.9, Jenkinson et al., 2002). BOLD runs were slice-time corrected to 0.591 seconds (0.5 of slice acquisition range 0 seconds–1.18 seconds) using 3dTshift from AFNI 20160207 (Cox & Hyde, 1997, RRID:SCR_005927). The BOLD time-series (including slice-timing correction when applied) were resampled onto their original, native space by applying the transforms to correct for head-motion. These resampled BOLD time-series will be referred to as preprocessed BOLD in original space, or just preprocessed BOLD. Several confounding time-series were calculated based on the preprocessed BOLD: frame-wise displacement (FD), DVARS, and three region-wise global signals. FD was computed using two formulations following Power (absolute sum of relative motions, Power et al. (2014)) and Jenkinson (relative root mean square displacement between affines, Jenkinson et al. (2002)). FD and DVARS are calculated for each functional run, both using their implementations in Nipype (following the definitions by Power et al., 2014). The three global signals are extracted within the CSF, the WM, and the whole-brain masks. Additionally, a set of physiological regressors were extracted to allow for component-based noise correction (CompCor, Behzadi et al., 2007). Principal components are estimated after high-pass filtering the preprocessed BOLD time-series (using a discrete cosine filter with 128 seconds cutoff) for the two CompCor variants: temporal (tCompCor) and anatomical (aCompCor). tCompCor components are then calculated from the top 2% variable voxels within the brain mask. For aCompCor, three probabilistic masks (CSF, WM, and combined CSF+WM) are generated in anatomical space. The implementation differs from that of Behzadi et al. in that instead of eroding the masks by two pixels on BOLD space, the aCompCor masks are subtracted a mask of pixels that likely contain a volume fraction of GM. This mask is obtained by dilating a GM mask extracted from the FreeSurfer’s aseg segmentation, and it ensures components are not extracted from voxels containing a minimal fraction of GM. Finally, these masks are resampled into BOLD space and binarized by thresholding at 0.99 (as in the original implementation). Components are also calculated separately within the WM and CSF masks. For each CompCor decomposition, the k components with the largest singular values are retained, such that the retained components’ time-series are sufficient to explain 50% of variance across the nuisance mask (CSF, WM, combined, or temporal). The remaining components are dropped from consideration. The head-motion estimates calculated in the correction step were also placed within the corresponding confounds file. The confound time-series derived from head-motion estimates and global signals were expanded with the inclusion of temporal derivatives and quadratic terms for each (Satterthwaite et al., 2013). Frames that exceeded a threshold of 0.5 mm FD or 1.5 standardized DVARS were annotated as motion outliers. All resamplings can be performed with a single interpolation step by composing all the pertinent transformations (i.e., head-motion transform matrices, susceptibility distortion correction when available, and co-registrations to anatomical and output spaces). Gridded (volumetric) resamplings were performed using antsApplyTransforms (ANTs), configured with Lanczos interpolation to minimize the smoothing effects of other kernels (Lanczos, 1964). Non-gridded (surface) resamplings were performed using mri_vol2surf (FreeSurfer).

Many internal operations of fMRIPrep use Nilearn 0.6.2 (Abraham et al., 2014, RRID:SCR_001362), mostly within the functional processing workflow. For more details of the pipeline, see the section corresponding to workflows in fMRIPrep’s documentation.
